# Etiology and Demographic Distribution of Odontogenic Abscesses in the Maxillofacial Area in Patients Over 18 Years of Age: A Five-Year Retrospective Study

**DOI:** 10.7759/cureus.59334

**Published:** 2024-04-30

**Authors:** Yanko G Yankov, Simeon Dimanov, Nikolay I Nikolaev, Lyuben Stoev, Ralitsa V Yotsova, Martina Stoeva

**Affiliations:** 1 Clinic of Maxillofacial Surgery, University Hospital "St. Marina", Varna, BGR; 2 Department of General and Operative Surgery, Medical University "Prof. Dr. Paraskev Stoyanov", Varna, BGR; 3 Department of Oral Surgery, Medical University of Varna, Varna, BGR; 4 Department of General and Clinical Pathology, Forensic Medicine and Deontology, Medical University of Varna, Varna, BGR

**Keywords:** purulent infection, drainage, incision, tooth, epidemiology, maxillofacial surgery, oral surgery, neck, head, abscess

## Abstract

Introduction

Despite the constant development of medicine and the increasing accessibility to medical specialists, in the first quarter of the 21^st^ century, odontogenic abscesses remain one of the leading causes of emergency hospitalization in maxillofacial surgery clinics. Because of the serious and lethal complications that this type of suppurative infection can lead to if not treated promptly, there is a need for constant updating of the knowledge of its origin, which is precisely what is addressed in this original article.

Materials and methods

It reports on a retrospective study conducted over a five-year period (2018-2023), during which 705 patients aged 18 years and older with a confirmed diagnosis of odontogenic soft tissue abscess of the head and neck underwent emergency surgery.

Results

The average age of the patients studied was 41 years, with the oldest being an 82-year-old woman. The proportion of males in the study population was higher - 54.18%. Young patients (18-44 years) were the most affected, with a total of 364 patients (213 males and 151 females), while the proportion of old people (75 years of age and older) was the lowest, with a total of 15 patients, including seven males and eight females. The first molars of both jaws (16, 26, 36 and 46) were the cause of the suppurative bacterial infection in the highest number among our study patients - 208 out of 705 (29.5%). Central incisors (teeth 11, 21, 31 and 41) were the least frequent direct cause of odontogenic infection, accounting for only 17 cases out of 705 (2.41%).

Discussion

The most logical reason for the decrease in the number of patients with odontogenic abscesses with increasing age is tooth loss in older individuals. Our study confirmed the knowledge that the first mandibular molars are the most common teeth leading to the formation of purulent exudate in the adjacent mandibular soft tissues. However, in contrast to the well-known fact for the maxilla that canines are the most frequent etiologic factor for the occurrence of odontogenic abscesses, we conclude that again the first molars (teeth 16 and 26) outnumber the other teeth of the maxillary dentition, with canines outnumbering only incisors. The teeth of the lower jaw are the cause of more than twice as many exudative infections as those of the upper jaw - the ratio between them is 2.54:1.

Conclusions

Knowledge of odontogenic abscesses - their demographic distribution, frequency and etiology, their diagnosis and treatment - is the basis for the prediction and treatment outcome of these diseases, mainly affecting young people. Their treatment is both surgical in order to evacuate the suppurative focus, and antibacterial.

## Introduction

Despite easy access to dental care and antimicrobial treatment in the 21^st^ century, odontogenic infections remain a serious clinical problem today. Decreased immune capabilities of the macro-organism, virulence of the causative microorganism and lack of adequate treatment lead to the spread of localized odontogenic infections to the deep maxillofacial spaces [1]. Odontogenic inflammations of the head and neck, if not treated promptly and adequately, can lead to life-threatening complications including airway obstruction, diffuse inflammatory processes (phlegmon), necrotizing fasciitis, purulent meningitis, cerebrospinal abscess, mediastinitis, sepsis and even end lethally [2,3].

Purulent inflammation in the maxillofacial region spreads in a known way - by the path of least tissue resistance [4,5]. Thus the infection passes from the spongiosa through the cortical plate of the jaw, into the periosteum, and thence into the soft tissues - initially the gingiva, and then along and through the fasciae of the head and neck [6]. In the lower jaw, a key factor for the localization and distribution of the inflammatory exudate is also the point of arising of the mylohyoideus muscle at the mylohyoid line. The line ends projectionally in the region of the roots of the first lower molar. This predisposes purulent inflammations originating from the second and third lower molars to spread into the submandibular space, and those from the medial standing teeth to spread into the sublingual space [7].

The purpose of this original article is to analyze the age-sex demographic distribution of patients with odontogenic abscesses and to determine the frequency of involvement of specific teeth of the human permanent dentition in the initiation of the inflammatory process, and thus to contribute to the understanding of these features in the current medical literature.

## Materials and methods

This original article examines a retrospective study conducted over a five-year period, from the beginning of 2018 to the end of 2023. During this time at the Clinic of Maxillofacial Surgery, University Hospital St. Marina (Varna, Bulgaria), 1176 patients with inflammatory diseases of the head and neck aged 18 years and older were hospitalized on an emergency basis and operated on. In 471 of them, the infection was of non-odontogenic origin, and in the remaining 705 patients it was odontogenic.

In this manuscript, we review and analyze only those 705 patients in whom the infection was confirmed to be odontogenic in nature. In all of them, the diagnosis of odontogenic abscess was made in two consecutive stages: during the first stage, the diagnosis of abscess was made, and during the second stage, it was confirmed that the origin of the same was odontogenic.

During the first stage, the diagnosis of an abscess was made on physical examination by a maxillofacial or oral surgeon, during which definite palpation evidence of a purulent infiltrate in the affected soft tissue area was found and described, and the overlying skin and/or mucosa was edematous, hyperemic, painful and with elevated local body temperature, which was recorded in patients' records.

The diagnosis of odontogenic abscess (second stage) was based both on the history of a painful tooth before the patients visited the facility and on the examination, which revealed painful percussion in a tooth or tooth roots. Orthopantomography (OPG) or computed tomography (CT) of the head and neck confirming the presence of dental pathology was performed in all patients prior to hospitalization as a routine procedure for diagnostic purposes, which is the direct cause of the formation of the purulent focus (entryway of infection) - periapical diffuse or localized chronic periodontitis, acute periodontitis, retained tooth roots, exacerbated odontogenic cysts, the first manifestation of which is the reason for patients to seek medical help, and exacerbated periodontal pockets.

The presence of abscess in all 705 patients was confirmed during operative treatment in incision and drainage volume, in which different amount of pus was evacuated and material was collected from the purulent focus and sent for microbiological examination and preparation of antibiogram by irrigating sterile gauze swab in the suppuration site.

Inclusion criteria were: the patients were 18 years of age or older, had been hospitalized and operated on, had a variable amount of pus evacuated during the incision, had definite anamnestic, physical and imaging evidence of odontogenic origin of the infection and physical and imaging evidence that the purulent focus was limited (abscess type).

Exclusion criteria were: patients under 18 years of age and those without definite history, physical and imaging evidence of an odontogenic cause of infection.

The information of all patients meeting the inclusion criteria was collected and tabulated in Microsoft Excel spreadsheets. The calculations in this study were performed using the SPSS v28 (IBM Corp., Armonk, NY, USA) program. The tables were created in "Microsoft Word" and the figures in "Microsoft Excel". "Windows" 7.0 (2010) software was used for all programs.

## Results

The mean age of 705 patients with odontogenic abscesses in the study population who were 18 years of age or older was 41±15.9 years, and the oldest patient was 82 years of age (Table 1).

**Table 1:** Age and sex distribution of the studied patients with odontogenic abscesses

Sex	Number	Percentage distribution (%)	Arithmetic mean	Median	Standard deviation	Minimum age	Maximum age
Men	382	54.18	37	35	13.82	18	81
Women	323	45.82	43	43	11.48	18	82
Total	705	100	41	37	15.9	18	82

**Figure 1:**
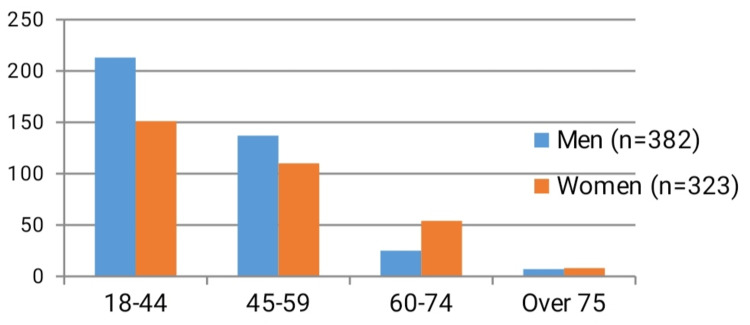
Age distribution of 705 patients with odontogenic abscesses studied

**Figure 2:**
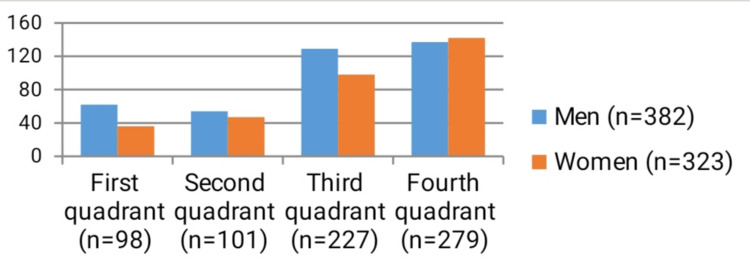
Distribution of patients with odontogenic abscesses in relation to the affected jaw segment

Table 1, Table 2, Figure 1 and Figure 2 show the distribution of the study group patients with odontogenic abscesses by sex, age groups and which jaw segment was affected by the disease.

The males of the study group dominated the females in the ratio of 1.18:1 (54.18%:45.82%). For convenience, we used the classification for demographic distribution of patients’ age groups according to the division that the World Health Organization (WHO) uses - young age (up to 44 years), middle (mature) age (45-59 years), elderly (60-74 years) and old people (over 75 years). The most affected patients were in the age range of 18-44 years, i.e., those in young age - a total of 364 patients (213 males and 151 females). The second most frequent were middle (mature) aged patients (between 45 and 59 years) - 247 patients (137 males and 110 females). Patients in the elderly group (between 60 and 74 years of age) had the third highest incidence with 79 patients (25 men and 54 women). The smallest proportion was of those aged 75 years and older (old people), with a total of 15 patients - seven men and eight women (Figure 1).

In the patients described, there was no anamnestic, physical or imaging evidence of the simultaneous presence of more than one dental causative agent of suppurative inflammation. The largest number of patients with an etiologic factor for odontogenic abscess caused by a tooth from the fourth maxillary quadrant (teeth 41-48), was 279 (137 males and 142 females). The second largest number of patients with odontogenic abscess caused by a tooth in the third quadrant (teeth 31-38) was 227 (129 men and 98 women). Patients with the etiological factor for suppurative inflammation tooth in the second quadrant (teeth 21-28) are the third most frequent - 101 people (54 men and 47 women). The smallest proportion of patients with odontogenic abscess caused by a tooth of the first quadrant (teeth 11-18) was 98 people (62 men and 36 women) (Figure 2).

It is noteworthy that the first molars (16, 26, 36 and 46) were the most frequent cause of the purulent bacterial infection in our study patients - 208 cases out of 705 (29.5%). The first teeth (central incisors - 11, 21, 31 and 41) of the maxillary quadrants were the least frequent cause of infection, with only 17 cases out of 705, representing 2.41% (Table 2).

**Table 2:** Distribution by number and jaw quadrants of teeth - etiological causes of head and neck abscesses in the studied patients

First quadrant (n=98)								Second quadrant (n=101)							
18	17	16	15	14	13	12	11	21	22	23	24	25	26	27	28
10	11	28	21	11	9	5	3	4	8	7	12	21	29	13	7
Fourth quadrant (n=279)								Third quadrant (n=227)							
48	47	46	45	44	43	42	41	31	32	33	34	35	36	37	38
18	35	79	69	60	8	7	3	7	7	13	54	40	72	16	18

## Discussion

The course of abscesses and the outcome of the disease, not only of the head and neck but also in general, depend on the immune status of the patient, the type and virulence of the microorganisms and the treatment performed [8]. Treatment of purulent odontogenic infections aims at evacuation of the purulent focus by incision and drainage of the abscess cavity, possibly removal of the source of infection, which in this case is the diseased tooth; other treatment options include oral or parenteral antibacterial therapy [9]. In our routine surgical practice, we do not always remove the source of infection during the incision of the abscess itself. In cases where the tooth can be preserved after healing, we leave it and, after the acute inflammation subsides, refer the patient to a dentist for further treatment with the aim of preservation.

Early diagnosis and treatment of odontogenic infections is of utmost importance for the rapid recovery of the patient, reduction of hospital stay and prevention of the risk of severe conditions. Systemic treatment includes an antibiogram and a selection of appropriate antibacterial treatments according to the results which it provides to the clinician [10]. Antibiogram material is taken during abscess drainage to determine the bacterial causative agents, their sensitivity to specific groups of antibacterial drugs and to determine the minimum inhibitory concentration (MIC). MIC is the lowest concentration of antibiotic that inhibits bacterial growth [11]. Its determination helps in prescribing the effective drug dose of the antibacterial agent [12]. In our clinical practice, we prescribe antimicrobial therapy empirically, even before the incision of the abscess, and only after the production of the antibiogram, which takes several days (usually two to four), we replace all or only some of the drugs with others if necessary.

Nonsteroidal anti-inflammatory drugs (NSAIDs) and paracetamol are used for adjunctive therapy. A number of clinical and paraclinical parameters such as body temperature, arterial blood pressure, leukocytosis, C-reactive protein (CRP) and procalcitonin (PCT) are monitored [13]. Standard diagnostic radiological investigations include ultrasonography and scanner with intravenous administration of contrast material, which helps to determine the exact localization of the process and to differentiate it from other inflammatory diseases such as suppurated cysts and tumors, acute and exacerbated chronic lymphadenopathies [14]. However, the main criteria for the diagnosis of odontogenic infection are the history of tooth pain and the detection of a diseased tooth on physical examination. Practice shows us that the detection of a diseased tooth is not always possible on physical examination, which requires imaging studies, most often sectoral photographs and OPG, on which we most often find residual roots, chronic diffuse or localized periapical periodontitis, periodontal pockets, cyst-like formations.

Prabhu and Nirmalkumar described a study of 1034 patients with neck infections and found that in 78.43% of all patients studied, the cause of the disease was odontogenic [15]. Statistics from our study showed that 705 out of 1176 patients had odontogenic cause for suppurative inflammation, which was 59.95%. The reason for the difference between the two studies is most likely due to the fact that while the study by Prabhu and Nirmalkumar analyzed only patients with neck infections, we also considered those with inflammation involving not only the cervical region but also the head. Another possible reason for the difference, we suggest, is the fact that their study examined patients of all ages, from 21 days to 96 years, whereas we analyzed only adult patients 18 years of age and older.

The etiology of odontogenic infections is most often related to the entry of pathogenic microorganisms into the soft tissues of the head and neck from necrotic dental pulp, acute and chronic periodontitis, periodontal pockets, exacerbated odontogenic cysts, and pericoronitis. According to the same study by Prabhu and Nirmalkumar, the most common cause of abscesses and phlegmons in the maxillofacial region is necrotic pulp of the molars and less commonly of the premolars and incisors [16]. Periapical periodontitis is an etiological factor in about 20-30% of cases [16]. According to the authors, odontogenic abscesses have the most frequent localization around the mandible. Our study proves this statement - the ratio of diseased teeth, directly responsible for the infection, on the mandible to those on the maxilla is 506:199, i.e., 2.54:1 (71.77:28.23%). Similar results were described by Shah et al. whose study reported that in suppurative dental infections, mandibular teeth were the more common etiological factor (73.5%) compared to maxillary teeth (26.5%) [17].

According to the study by the same authors, the most frequently affected mandibular tooth is the right first molar (tooth 46) - 28% of cases - and the maxillary tooth is the right canine (tooth 13) - 16% of cases [17]. Our study confirms that the tooth most frequently responsible for dental infections not only in mandibular teeth but also in general is tooth 46, but in maxillary teeth, the most frequent causative tooth is the upper left first molar (tooth 26). According to our study, upper canines ranked only after premolars and molars as an etiologic factor for odontogenic suppurative inflammation, but were superior to maxillary incisors.

The sex demographic distribution of patients with odontogenic abscesses and phlegmons was described and analyzed in 2023 by Pucci et al. and according to them, the sex ratio was 54.4% for males and 45.6% for the female population [18]. According to our present study, the sex ratio of males:females is almost no different from that described by them, 54.18%:45.82%, thus proving that purulent inflammatory diseases of the head and neck of odontogenic origin occur more frequently in males than in females. The world literature lacks data explaining this phenomenon, and the most probable reason for this is the heterogeneity of the samples of patients that the conducted studies examined and analyzed.

According to our study, the incidence of odontogenic infections decreases with advancing age (Figure 1). Patients in the group 18-44 years were the most affected, and their relative proportion gradually decreased with increasing age. Patients over the age of 75 years are the least affected and the most logical reason for this is that with age people lose more and more teeth due to a number of reasons [19]. Another explanation could be the average life expectancy in Bulgaria. By 2021, it was 74.8 years on average. After 2021, due to the Covid-19 pandemic, the same decreased to 74.64 years [20]. This in fact shows why with increasing age, the incidence of infections of odontogenic origin decreases.

Limitations

As limitations of this article, it can be noted that it covers only a five-year time interval during which the patients were studied, that it considers patients who were hospitalized in only one clinic of one hospital, the fact that it does not study the pediatric population, the nature of the study is retrospective, and there is a lack of statistical analysis in the study.

## Conclusions

Odontogenic inflammatory diseases of the head and neck are more common in younger individuals and their incidence decreases with age, a fact that is most likely due to the loss of teeth in people with age. They are more common in males, for which there is no conclusive evidence in the world literature. Our study confirms that the most common cause of their occurrence in patients over 18 years of age is the first lower right molar. Of the maxillary teeth, the upper left first molar is the most common cause of the formation of purulent exudate in the adjacent soft tissue spaces of the jaw.
